# Validation of a Commercial Glanders ELISA as an Alternative to the CFT in International Trade of Equidae

**DOI:** 10.3389/fvets.2021.628389

**Published:** 2021-02-16

**Authors:** Mandy Carolina Elschner, Falk Melzer, Harisankar Singha, Saqib Muhammad, Ian Gardner, Heinrich Neubauer

**Affiliations:** ^1^Friedrich-Loeffler-Institut (FLI), Federal Research Institute for Animal Health, Institute of Bacterial Infections and Zoonoses, Jena, Germany; ^2^Indian Council of Agricultural Research, National Research Centre on Equines, Hisar, India; ^3^Department of Clinical Medicine and Surgery, University of Agriculture, Faisalabad, Pakistan; ^4^Department of Health Management, Atlantic Veterinary College, University of Prince Edward Island (UPEI), Charlottetown, PE, Canada

**Keywords:** glanders, *Burkholderia mallei*, serology, CFT, western blot, ELISA, sensitivity, specificity

## Abstract

Glanders, caused by *Burkholderia* (*B*.) *mallei* is a notifiable zoonotic disease in equidae. For international trade and movement of equids, certificates of negative serological test results for antibodies against *B. mallei* are required. To date, the complement fixation test (CFT) is the mandatory test to issue these health certificates. The CFT is difficult to standardize and, due to its poor specificity, often leads to false-positive reactions resulting in trade restrictions with considerable financial consequences. In the present study, the new ID Screen Glanders Double Antigen Multispecies ELISA (GLANDA- ELISA) (IDvet, Grabels, France) was evaluated using 400 negative and 370 glanders positive field samples of equidae. The GLANDA-ELISA was significantly more specific (99.8%) than the CFT (97.0%). Considering the comparable sensitivities of CFT (96.5%) and ELISA (98.1%), this new GLANDA-ELISA test appears a suitable confirmatory test and a realistic alternative for serological testing of horses for trade or movement.

## Introduction

*Burkholderia* (*B*.) *mallei* is the causative agent of the zoonosis glanders of equidae. The horse, in which the infection is often chronic and asymptomatic, poses a risk for reintroducing the infection into areas where it had been eradicated. Mandatory testing and certification of freedom from glanders in the single animal before movement are intended to prevent spread of disease. Although the disease has been eradicated in most European and North American countries, regular outbreaks are reported in countries of South America and Asia (1–7).

The “gold (reference) standard” of glanders diagnostics is the isolation of *B. mallei* from clinical samples. However, poor sensitivity and considerable time consumption makes the cultural isolation a less preferred method for glanders diagnosis. Therefore, indirect methods such as the mallein test (allergic hypersensitivity test) and complement fixation test (CFT) are available. The mallein test is not accepted for trade testing and is generally not recommended because of animal welfare concerns. Furthermore, malleinised animals may undergo seroconversion and then show positive reactions in additional diagnostic methods, like CFT (8). However, the mallein test may be useful for glanders eradication in remote endemic regions (6, 9–11). In particular, CFT has been used for decades for surveillance, confirmation of outbreaks and trade testing; it is also compulsory based on recommendations of the World Organization for Animal Health (OIE) (8). Studies have shown that the CFT is very sensitive, but unfortunately it produces a considerable number of false-positive results (12–14), which then lead to serious restrictions on international trade. CFT also has the technical disadvantages like it is labor-intensive and difficult to standardize because there are no international standard sera available. The test antigens and the testing protocols used in the laboratories differ, which can have significant influence on the results (15–18). Thus, attempts have been made to develop alternative tests with comparable sensitivity and higher specificity for years. Methods such as Western blot (WB) and various ELISAs have already been developed and some were already validated according to the principles of validation of diagnostic assays for infectious diseases (14, 18–20). For example, a Western blot procedure showed a significantly higher diagnostic specificity when compared to CFT and currently is used in Germany as a confirmatory test for glanders (13, 14, 21). Although there are tests that are comparable to CFT in terms of sensitivity and specificity, but are not yet been commercially available and officially approved by competent authorities. The aim of the study was to evaluate whether the new GLANDA-ELISA, developed and commercialized by IdVet (IDvet, Grabels, France) could be accepted as an alternative method to CFT for the examination of equidae for trade certificates.

## Methods

### Sera

True-positive field serum samples (*n* = 370) were collected from equids during glanders outbreaks in Pakistan 2007, 2016–2020 (*n* = 236), and India (*n* = 132) in 2016 and 2017. Two samples originated from horses found positive by CFT, WB, and PCR in Germany, in 2006 and 2014. The samples from India and Pakistan originated from animals in which the infection was either confirmed by clinical signs and CFT (*n* = 117), or by *B. mallei* isolation or molecular detection of *B. mallei* using real-time PCR (*n* = 251) as recommended by OIE (8). The animals were considered to be “clinically positive” on the basis of clinical signs consistent with glanders, and the fact, that they were detected during a cultural confirmed glanders outbreak in its population including close contact to infected animals. The whole collection consisted of 338, 25, and 7 sera from horses, mules, and donkeys, respectively.

True-negative samples (*n* = 400), were collected in Germany, which is officially free from glanders. All samples were collected non-randomly during routine testing by CFT for trade or movement.

### Ethics Statement

For this study, no ethical approvals were required. All blood samples were routinely collected for prescribed diagnostic purposes or official monitoring studies and subsequently made available to this study.

### Description of the Tests

The characteristics of the 3 tests compared in this study are given in Table 1.

**Table 1:** Name of the test, test developer, and antigens used in the 3 assays compared.

**Test**	**Developer/protocol used**	**Antigen used**
ID Screen Glanders Double Antigen Multispecies ELISA (GLANDA ELISA)	IDvet, Grabels, France	Recombinant antigen and a second recombinant protein of *B. mallei*
Western blot (WB)	FLI, Germany (13)	Crude preparations of LPS antigen of 3 different *B. mallei* strains Bogor, Mukteswar, and Bahrain1
Complement fixation test (CFT)	OIE-Manual (8)	Malleus-CFT antigen; Ccpro GmbH (Oberdorla, Germany) prepared from *B. mallei* strain Ivan-NCTC 10230

### Complement Fixation Test (CFT)

CFT was performed in a 96 well “U” bottom plate as described in the OIE manual (8). Serum samples (1:5 diluted) were tested with Malleus CFT antigen (Ccpro GmbH, Germany) and 5 complement haemolytic units-50% of guinea pig complement (Institute Virion/Serion GmbH, Germany). Serum, complement and antigen were mixed and incubated overnight at 4°C. Then a 2% suspension of sensitized sheep red blood cells (Institute Virion/Serion GmbH) was added, and after 45 min at 37°C the plate was centrifuged for 5 min at 600 g. A sample that produced 100% haemolysis at the 1/5 dilution was classified as negative, 25–75% haemolysis as suspicious, and no haemolysis was positive.

### Western Blot (WB)

The Western blot has been described in detail in previous publications (13, 14). Briefly, LPS-containing antigens of *B. mallei* strains Bogor, Mukteswar, and Bahrain1 were transferred on a nitrocellulose membrane. For the immunoblot analysis the serum samples at a dilution of 1:50 were incubated with the membranes. Anti-horse conjugate labeled with alkaline phosphatase (Sigma, Munich, Germany) was used at a dilution of 1:5,000 as the secondary antibody. Detection was performed by a ready to use NBT-BCIP® staining system (Sigma). The WB was scored positive, if the banding pattern of the LPS ladder was clearly visible within the region of 20–60 Kilo Dalton (kDa).

### ID Screen Glanders Double Antigen Multispecies ELISA (GLANDA-ELISA)

The GLANDA-ELISA (IDVet, Grabels, France) was provided as a ready-to-use kit and was performed according to the manufacturer's instructions. The kit contained recombinant *B. mallei* antigen-coated plates with individual 8-well strips, positive and negative controls, 10x concentrated recombinant purified *Burkholderia mallei* protein antigen-HRP conjugate, dilution buffers, 20x wash concentrate, TMB substrate solution, and stop solution (0.5 M). Briefly, 90 μl dilution buffer and 10 μl of the serum samples were added to an antigen-coated well. After 45 ± 5 min incubation at 21 ± 5°C, wells were emptied, washed 3 times and incubated with the conjugate for 30 ± 3 min at 21 ± 5°C. Washing was repeated 3 times. Then 100 μl of conjugate (1x) were added and after incubation for 30 ± 3 min the 3-fold washing procedure was repeated. Finally, 100 μl of the substrate solution were added and after 15 ± 2 min incubation at 21 ± 5°C the reaction was stopped by 100 μl of the stop solution and OD 450 nm value was recorded. S/P% values were calculated and samples with S/P% <70% were considered as negative, and ≥70% as positive.

### Statistical Analysis

Calculations including 95% confidence interval (CI) were based on standard formulas (22) and done using MedCalc version 13.1.0.0 (https://www.medcalc.org/). Sensitivity and specificity of test pairs were compared with McNemar's test for correlated proportions with *P* < 0.05 considered statistically significant.

## Results

### Diagnostic Specificity (DSp) and Diagnostic Sensitivity (DSe)

The data and calculations for DSp and DSe are given in Table 2. When comparing all tests in pairs between the three tests, no significant differences in DSe were found. However, when comparing the results for the true-negative samples, the GLANDA-ELISA was highly significantly more specific than the CFT (*P* = 0.003) and the WB (*P* = 0.035) also showed a significant higher DSp than the CFT.

**Table 2:** Diagnostic specificity (DSp) of 400 tested true negative samples, and diagnostic sensitivity (DSe) of 370 tested true positive samples with respective 95% CI; TN-true negatives, TP-true positives, FN-false negatives, FP-false positives.

**DSp testing 400 true-negative samples**				
	**FP**	**TN**	**DSp%**	**CI 95%**
GLANDA-ELISA	1	399	99.8	98.6–100.0
WB	3	397	99.2	97.8–99.8
CFT	12	388	97.0	94.8–98.4
**DSe testing 370 true-positive samples**				
	**FN**	**TP**	**DSe%**	**CI 95%**
GLANDA-ELISA	7	363	98.1	96.1–99.2
WB	10	360	97.3	95.1–98.7
CFT	13	357	96.5	94.1–98.1

### Likelihood Ratios and Predictive Values

Table 3 shows the positive and negative likelihood ratio values of the tests, as well as the positive and negative predictive values for scenarios of 0.01 and 3% disease prevalence, which were considered to be the typical or worst case prevalences in equid populations, respectively.

**Table 3:** Likelihood ratios for GLANDA-ELISA, CFT, and WB and predictive values in scenarios of 0.01 and 3% disease prevalence.

**Assay**	**GLANDA-ELISA**	**CI 95%**	**WB**	**CI 95%**	**CFT**	**CI 95%**
LR+	392.4	55.4–2779.3	129.7	42.0–400.6	32.2	18.4–56.2
LR–	0.02	0.01–0.04	0.03	0.01–0.05	0.04	0.02–0.06
**Prevalence 0.01%**						
PPV	4.68	0–98.0	1.20	0–47.5	0.32	0–15.4
NPV	100	99.5–100	100	99.5–100	100	99.5–100
**Prevalence 3%**						
PPV	93.8	76.0–99.6	79.0	59.5–92.0	49.9	34.4–65.3
NPV	99.9	99.4–100	99.9	99.3–100	99.9	99.3–100

*LR+, Positive Likelihood Ratio; LR–, Negative Likelihood Ratio; PPV, Positive Predictive Value; NPV, Negative Predictive Value*.

The frequencies of combinations of test results in known infected and non-infected populations are shown in Table 4.

**Table 4:** Frequency of combinations of test results in known infected and non-infected populations.

**Infected population (true-positives)**		**Non-infected population (true-negatives)**	
GLANDA-ELISA+ CFT+ WB+	348	GLANDA-ELISA– CFT– WB–	384
GLANDA-ELISA+ CFT– WB+	10	GLANDA-ELISA– CFT+ WB–	12
GLANDA-ELISA+ CFT+ WB–	4	GLANDA-ELISA– CFT– WB+	3
GLANDA-ELISA– CFT+ WB–	3	GLANDA-ELISA+ CFT– WB–	1
GLANDA-ELISA– CFT+ WB+	2		
GLANDA-ELISA– CFT– WB–	2		
GLANDA-ELISA+ CFT– WB–	1		
Total	370	Total	400

*Combinations of test results with zero counts are not listed in the table*.

## Discussion

The CFT test has been used for serological diagnosis of glanders for more than 100 years (23). In particular, its high sensitivity in combination with a consistent culling of test-positive equidae has contributed to the eradication of the disease in many regions of the world. However, the lack of specificity and the resulting obstacles to the trade of equids on the one hand, and the difficulties in standardization of the CFT on the other hand, have stimulated research for alternative serological methods (15–17). In addition to Western blot methods (13, 21, 24), several ELISA based on crude *B. mallei* antigen preparations and various recombinant proteins have already been developed and evaluated (14, 19, 20, 25, 26).

In the present study, the diagnostic accuracy of the commercially-available GLANDA-ELISA and the OIE-described CFT were compared on samples from equids of known infection status. Furthermore, the WB used presently as confirmatory test in the German approach for glanders serodiagnosis (21) was included in the comparison.

Based on the results of 400 negative serum samples, it was shown, that the GLANDA-ELISA was significantly more specific than the CFT. Previously published reports have shown that ELISAs can improve specificity. The specificity of an iELISA using HCP1 as single antigen was 99.6%, but sensitivity was only 95.3% (14). For all validation studies of serological tests for glanders, the procurement of positive samples is the greatest challenge. The acquisition of positive samples for this study was not only based on their serologically positive results, but was done by highly experienced veterinarians in Pakistan and India considering the epidemiologic situation, clinical investigations, and results of confirmatory microbiological tests. This approach was also used in a preliminary study (14) and allowed the number of true-positive samples to be increased and thus the precision in DSe estimates was increased. The evaluation of test sensitivity in this study revealed comparable data for CFT, GLANDA-ELISA, and WB. For international trade it is generally accepted that the most sensitive test has to be applied to avoid an introduction of infected animals into disease-free populations (27). The OIE specifications for a confirmative serological test for glanders are equal or higher sensitivity and higher specificity than for the CFT (8). Considering both sensitivity and specificity, the WB (97.3%, 99.2%) and the GLANDA-ELISA (98.1%, 99.8%) met these OIE requirements.

However, the CFT is still the prescribed method for trade purposes to certify individual animal free from glanders (8). The test requires experienced operators because performing the assay is demanding and the results depend on the quality of the antigen and procedures used, such as incubation conditions (15, 16). Study data confirmed that combinations of different tests can enhance the DSp of serological diagnosis i.e., CFT with the ELISA (*p* = 0.003) and CFT and WB (*p* = 0.035). In Germany, false-positive CFT results are ruled out by using the WB as a confirmatory test (21). The WB is very well-suited for individual animal testing but it is time consuming and restricted to few experienced laboratories. However, this study shows, that the GLANDA-ELISA can identify infected animals with high confidence and demonstrates the freedom from glanders in animals for movement. The reason for the very good test properties with regard to sensitivity and specificity might be the new double antigen approach of the GLANDA-ELISA, which is hitherto unique to glanders ELISAs. In particular, the rapid and simple testing protocol qualify the GLANDA-ELISA as a reliable method even for handling large number of samples in standard diagnostic laboratories.

## Data Availability Statement

The raw data supporting the conclusions of this article will be made available by the authors, without undue reservation.

## Ethics Statement

Ethical review and approval was not required for the animal study because for this study, no ethical approvals were required. All blood samples were routinely collected for prescribed diagnostic purposes or official monitoring studies and subsequently made available to this study. Written informed consent was obtained from the owners for the participation of their animals in this study.

## Author Contributions

ME, HS, and SM: collection, characterization of samples, and writing—original draft. ME: investigation. ME and IG: statistical data analysis. HN: supervision. ME, HS, SM, FM, IG, and HN: writing—review and editing. All authors contributed to the article and approved the submitted version.

## Conflict of Interest

The authors declare that the research was conducted in the absence of any commercial or financial relationships that could be construed as a potential conflict of interest.
